# 

**Published:** 1918-01

**Authors:** 


PUBLISHED MONTHLY. ATLANTA SUBSCRIPTION $1.00

### JOURNAL-RECORD OF MEDICINE

Successor to { Atlanta Medical and Surgical Journal, Established 1855. and Southern Medical Record, Established 1870. } 64th Year

Vol. LXIV ATLANTA, GA., JANUARY, 1917 No. 10

